# Brain Extracellular Space: From an Overlooked Dimension to Catalyst of a Novel Neuroscience Paradigm

**DOI:** 10.34133/cbsystems.0529

**Published:** 2026-03-04

**Authors:** Hongbin Han, Hui Dai, Leonor Serrano Lopes, Ruiqing Ni, Benjamin F. Combes, Yangjing Song, Hanbo Tan, Meng Xu, Hongfeng Li, Shuhong Lv, Zhaohe Yang, Tianzi Gao, Mengyu Zhang, Yang Shi, Jingjing Shao, Yanni Zhang, Wanyi Fu

**Affiliations:** ^1^ Institute of Medical Technology, Peking University Health Science Center, Beijing 100191, China.; ^2^ Department of Radiology, Peking University Third Hospital, Beijing 100191, China.; ^3^ Beijing Key Laboratory of Intelligent Neuromodulation and Brain Disorder Treatment, Beijing 100191, China.; ^4^ Department of Nuclear Medicine, Inselspital, Bern University Hospital, University of Bern, Bern 3010, Switzerland.; ^5^ Graduate School for Cellular and Biomedical Sciences, University of Bern, Bern 3012, Switzerland.; ^6^ Institute for Biomedical Engineering, Department of Information Technology and Electrical Engineering, ETH Zurich, Zurich 8093, Switzerland.; ^7^ Institute for Regenerative Medicine, University of Zurich, Zurich 8952, Switzerland.; ^8^ School of Pharmaceutical Sciences, Cheeloo College of Medicine, Shandong University, Shandong 250012, China.

## Abstract

Despite huge investment, therapies for brain disorders remain largely ineffective in clinical practice. Accumulating evidence indicates that this low translational success is closely linked to the long-standing overlook of the brain extracellular space (ECS) in preclinical research, clinical practice, and regulatory frameworks. After over 4 decades of scientific exploration, particularly with recent breakthroughs in imaging and quantitative measurement methods, it is timely to integrate the ECS into the current neuroscience framework. This paper investigates underlying determinants of low translational success of central nervous system drugs and therapeutic devices, reviews the historical and technical bottlenecks that lead to the neglect of ECS research, and emphasizes its transformative potential in reshaping therapeutic strategies. We propose incorporating the ECS into neuroscience research, clinical regulatory assessment, and medical education, thereby establishing a comprehensive paradigm that omits no physical space for precision therapeutics targeting brain disorders.

## Introduction

**Fig. 1.**
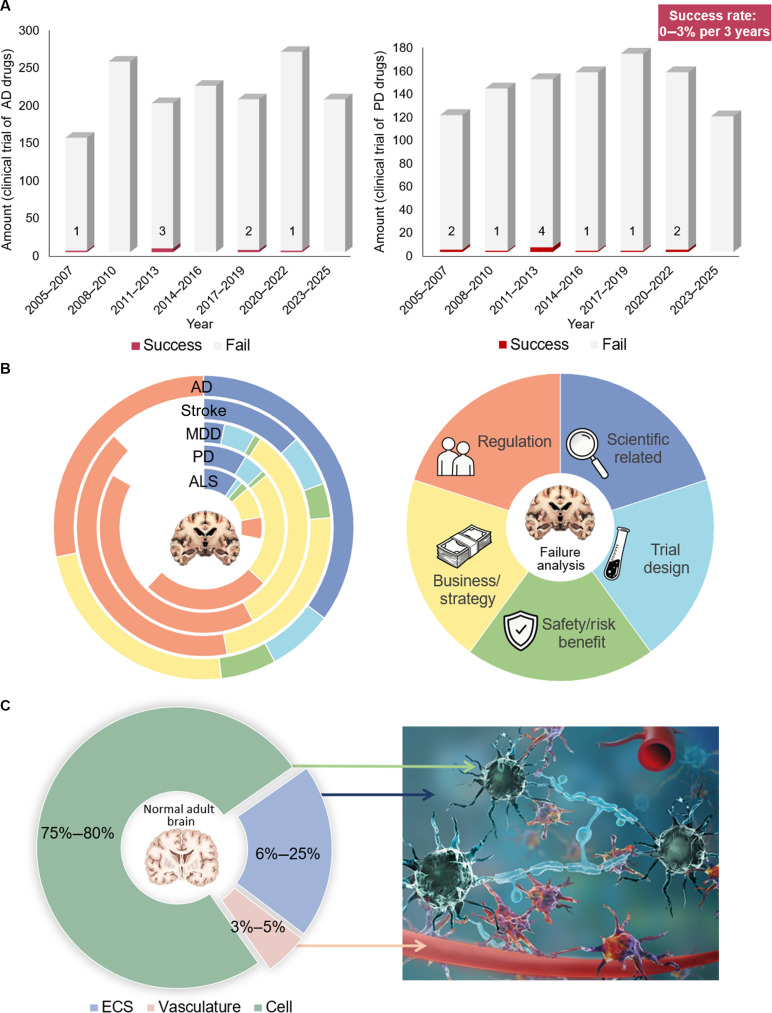
Failure analysis of CNS disorder therapeutics and therapeutic device development (2005–2025). (A) Number of registered clinical trials for AD and PD therapeutics on ClinicalTrials.gov from 2005 September 1 to 2025 September 1, with an overall average success rate of ~0 to 3% per 3-year interval. (B) Root causes of clinical trial failure for diverse CNS disorder therapeutics and therapeutic devices. Flaws in preclinical and clinical trial design are intrinsically linked to the neglect of the ECS. MDD, major depressive disorder; ALS, amyotrophic lateral sclerosis. (C) Volume fraction of distinct compartments in the normal brain. The ECS accounts for 6% to 25% of the total brain volume.

Over the past century, neuroscience research has centered on neurons, glial cells, and vascular networks. These endeavors have yielded milestone advances that have fundamentally redefined our understanding of brain function [1,2]. However, these advances have translated into only limited clinical success (Fig. 1A). Pharmaceutical and device companies have invested hundreds of billions of dollars in research against every major brain disorder, but, unfortunately, few achievements have proven definitely beneficial for patients suffering from the central nervous system (CNS) diseases. The mystery of human memory and emotion is still an open question; meanwhile, Alzheimer’s disease (AD) or cerebral stroke continues to cause disability and crushes human dignity.

In AD, for example, more than 400 phase III clinical trials have been conducted, but only a handful of antiamyloid antibodies have received conditional regulatory approval [3,4]. Even the high-profile agent aducanumab demonstrated only modest efficacy: Its reduction of amyloid burden did not translate into clear, durable clinical benefit, leaving its therapeutic value a subject of scientific debate [3]. A similar scenario applies to psychiatric medications and neuroprotective drugs targeting cerebral ischemic stroke (Fig. 1B) [5–7]. Recent findings regarding the brain extracellular space (ECS) reveal a theoretical structural flaw inherent in the existing research framework for neuroscience and the treatment of brain disorders: The critical role of the brain ECS has long been overlooked.

The brain ECS constitutes the core anatomical structure of the brain interstitial system (ISS) [8]. It is defined as irregular and tortuous microspaces constrained by the plasma membranes of various cells in the brain parenchyma (Fig. 1C). The ECS is primarily composed of the extracellular matrix (ECM) and interstitial fluid (ISF). Notably, the biochemical composition of the brain ECM differs substantially from that of other organs. It is rich in hyaluronic acid (HA) and proteoglycans yet lacks fibrous components such as collagen and fibronectin. This unique ECM scaffold is filled with ISF that exhibit heterogeneity across different brain regions. Basic constituents include water, ions, gaseous molecules, and organic molecules such as proteins, peptides, enzymes, neurotransmitters, and extracellular vesicles, along with glycoprotein chains anchored to the ECM [9,10]. The ECS serves not merely as a reservoir for substances; its contents communicate and exchange with the cerebrospinal fluid (CSF) in the subarachnoid space via multiple physical and physiological pathways, and, consequently, the ECS is regarded as a primary pathway for metabolic waste clearance in the brain [8]. The ECS also provides a pathway for the drugs to reach their target and exert effects once crossing the blood–brain barrier (BBB) [11].

Increasing evidence indicates that the ECS is not only a determinant of homeostatic regulation but also contributes to the onset, progression, and outcome of brain diseases [12–15]. However, the ECS has remained largely absent from prevailing frameworks for therapeutic design and efficacy evaluation. The ECS occupies approximately 6% to 25% of the living brain volume (Fig. 1C) [16–19]. However, a bibliometric analysis of the Scopus database indicates that among more than 2 million neuroscience publications, fewer than 9,000 explicitly focus on the ECS, accounting for only ~0.45% of the literature. This mismatch has created a structural blind spot and knowledge gap, which is insufficient to satisfy the stringent requirements of global regulatory agencies for comprehensive characterization of drug mechanisms of action and pharmacokinetic (PK) distribution profiles.

**Fig. 2.**
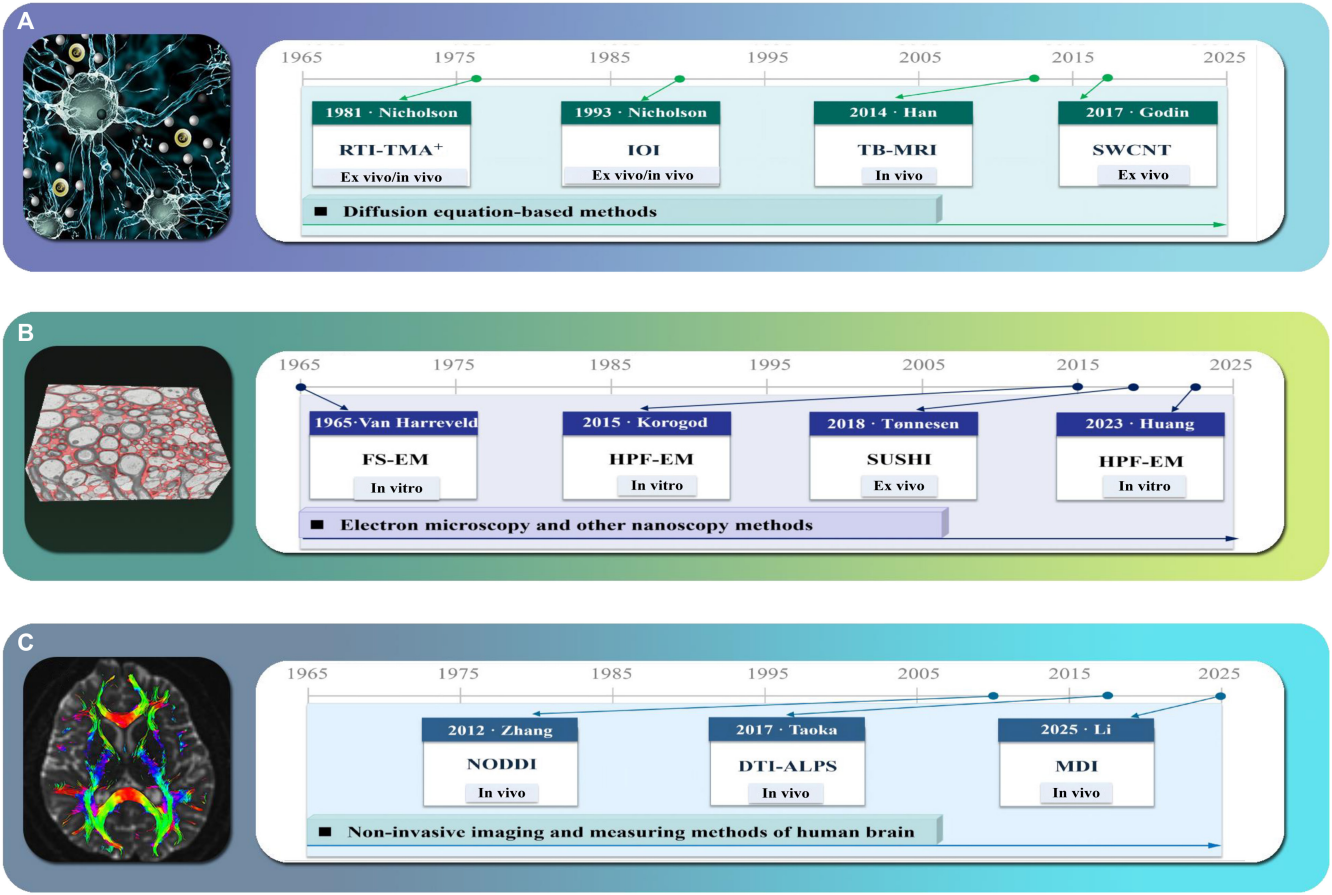
Chronological schematic of 3 technical approaches for 2 categories of ECS measurement techniques. Tracer-based methods are illustrated in (A), while the 2 technical approaches of label-free measurement methods are depicted in (B) and (C). (A) Diffusion parameters of the cerebral ECS were derived by solving the inverse problem of the advection–diffusion equation, encompassing primarily RTI-TMA^+^, IOI, SWCNTs, and TB-MRI. (B) Nanoscopy-based approaches, mainly including freeze substitution EM (FS-EM), high-pressure freezing EM (HPF-EM), and super-resolution shadow imaging (SUSHI). (C) Label-free methods are also termed noninvasive measurement techniques and primarily comprise NODDI, DTI-ALPS, and MDI.

Historically, the primary reason that neuroscientists have long neglected or even overlooked the ECS lies in the limitations of low-spatial-resolution imaging techniques (Fig. 2). Similarly, the structural characterization of the ECS demands rigorous, imaging-based validation, much similar to how Santiago Ramón y Cajal provided definitive evidence for the neuron doctrine through staining methods and anatomical illustrations [20]. Electron microscopy (EM) stands as the gold standard for visualizing nanoscale subcellular structures. However, early attempts to image the ECS by EM were unsuccessful, largely attributable to preimaging sample preparation protocols. Specifically, procedures such as chemical fixation, staining, and dehydration not only distort the native ultrastructure of brain tissue but also induce ECS collapse [21]. Recent breakthroughs in artificial intelligence (AI)-assisted cryo-EM and super-resolution optical imaging technologies now enable the visualization of the ECS’s true structure, just as Cajal did over a century ago in establishing the neuron doctrine [17,18].

The Food and Drug Administration (FDA) Modernization Act 2.0 has authorized in vitro systems and AI-enabled computational models as alternatives to replace animal testing [22], rendering the systematic integration of the ECS into CNS therapeutic research both urgent and necessary. If the ECS remains excluded, emerging “nonanimal” models is likely to reproduce existing blind spots in brain spatial organization and physiology. In the absence of ECS-informed fundamentals, AI simulations and in vitro platforms will be structurally limited in faithfully recapitulating and predicting drug distribution and action in the living brain microenvironment [8,23].

Integrating the ECS into the research and evaluation frameworks is not only a scientific necessity but also an increasingly explicit regulatory demand. This review dissects the historical and technical underpinnings behind the systematic overlook of the ECS from CNS research and therapeutics, explains how this neglect has contributed to the long-standing low translational efficiency of CNS therapeutics, and highlights the value of incorporating the ECS into future neuroscience and CNS therapeutic frameworks. Finally, we propose a new “cell–ECS–vasculature” integrated paradigm that explicitly embeds the ECS into translational research, clinical trials, regulatory evaluation, and medical education, thereby paving the way toward a structurally complete and quantitatively measurable foundation for precision treatment of CNS disorders, where no anatomical compartment is omitted.

## Imaging and Measurement Technologies of the Brain ECS

**Fig. 3.**
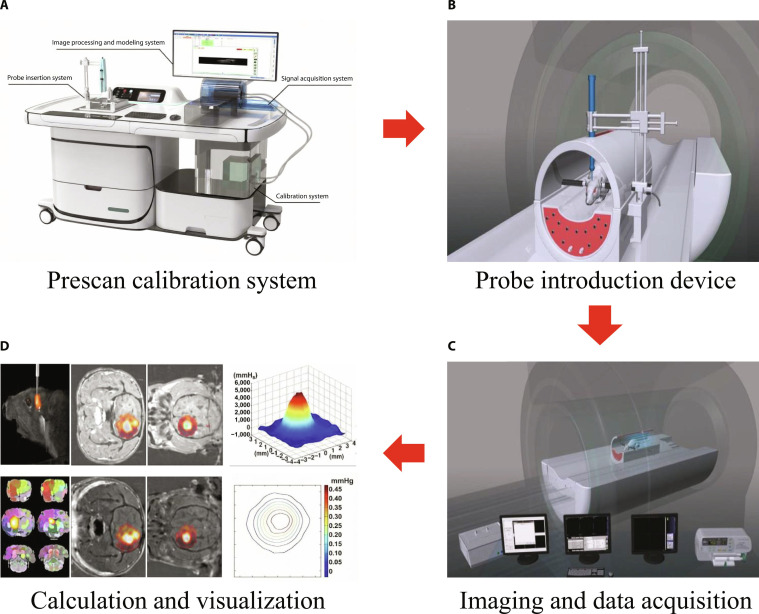
Schematic diagram of tracer-based ECS measuring methods (illustrated with tracer-based MRI). (A) Probe preparation and calibration process to verify the specific distribution of probes in the ECS and quantify the correlation between probe concentration and signal intensity. (B) Probe administration via ECS. (C) Signal acquisition of dynamic probe distribution in the brain ECS. (D) Modeling, calculation, and result visualization.

**Fig. 4.**
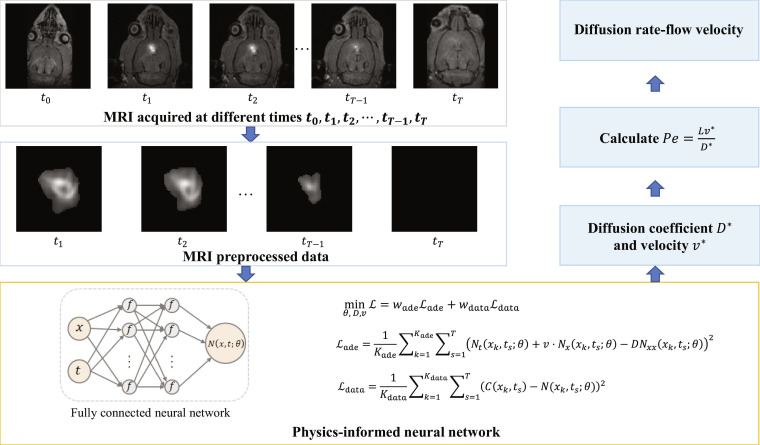
Flowchart of ECS substance transport modeling based on PINN. Time-series images are acquired and preprocessed to extract tracer distribution. A PINN with parameters 𝜃 takes spatial coordinates (𝑥) and time (𝑡) as inputs and is optimized to inversely solve for the diffusion coefficient (𝐷), and molecular velocity (𝑣) to calculate the Péclet number.

**Fig. 5.**
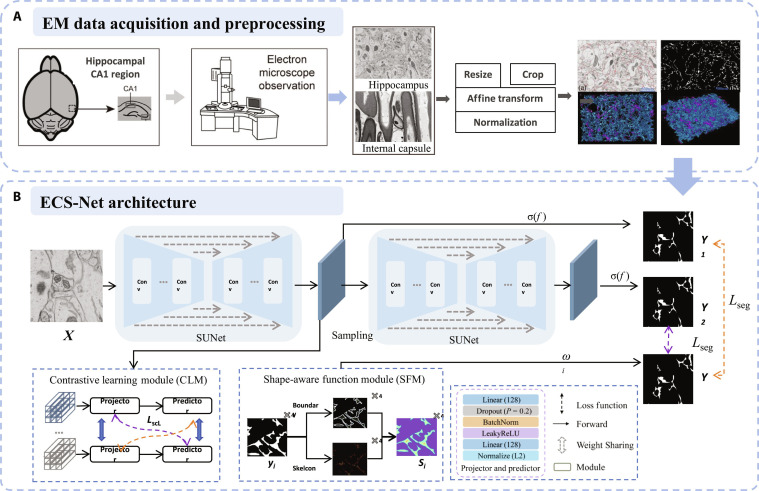
The EM-based ECS reconstruction procedure. (A) ECS EM data acquisition and preprocessing. (B) The ECS-Net architecture. The contrastive learning module maximizes the distance between category representations, and the shape-aware module improves the structural constraints for complex shapes.

**Fig. 6.**
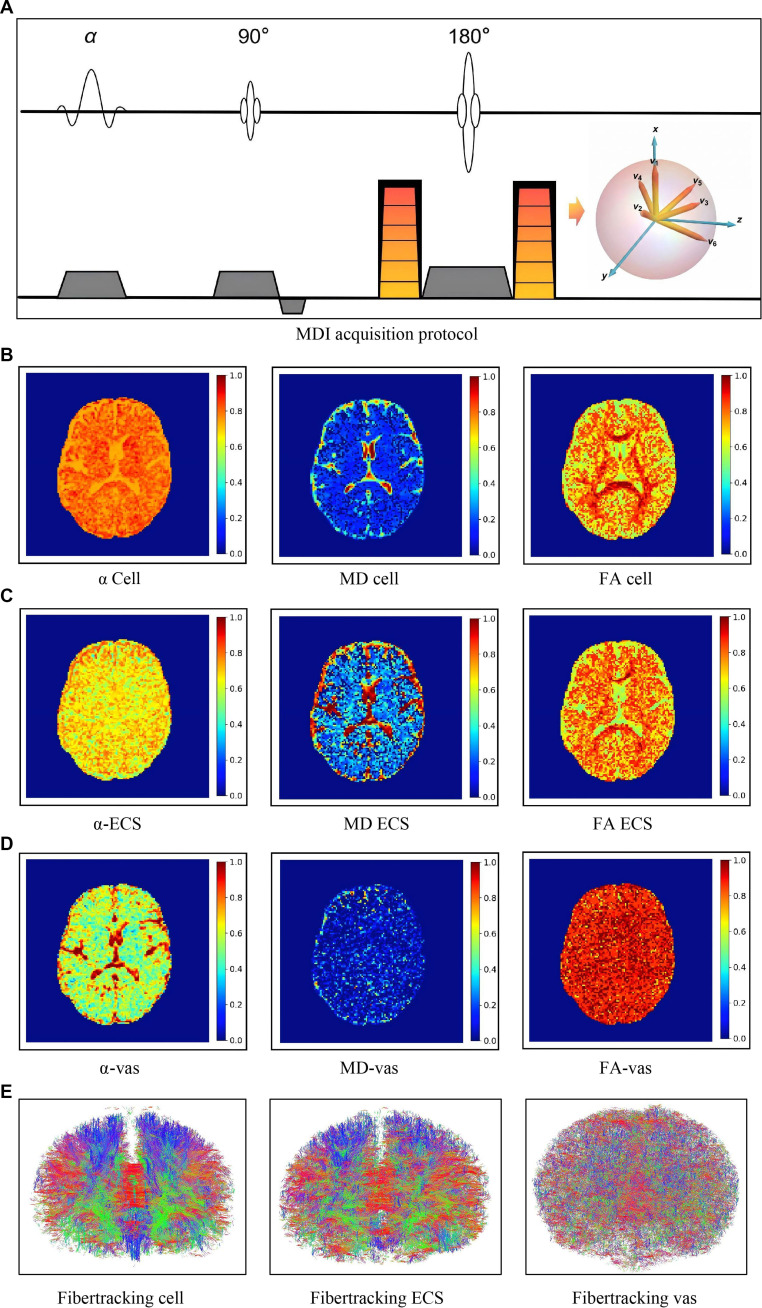
Schematic illustration of MDI. (A) MRI sequence design of MDI. (B) Biophysical parameter mappings of neural cells, including volume fraction (left), diffusion rate (middle), and fraction of anisotropy (right). (C) Mappings of ECS with the same parameters. (D) Mappings of microvasculature (vas) with the same parameters. (E) Whole-brain tractography of the 3 compartments.

The measuring and imaging methods of the brain ECS can be classified into 2 categories: tracer-based methods with probes and label-free methods with endogenous contrast (Table 1 and Fig. 2). Tracer-based methods introduce probes into the ECS to acquire spatiotemporal changes in their signals (Fig. 3) [16,24–26]. ECS biophysical parameters are then estimated by solving inverse problems derived from the diffusion models (see the “Measurement methods based on solving the inverse problem of the diffusion equation” and “Tracer-based optical imaging” sections and Fig. 4). Label-free methods distinguish between cells and the ECS without the need for any probes, for instance, by utilizing membrane staining to enhance cell–ECS contrast and derive ECS structural characteristics (see the “Electron microscopy” section and Fig. 5) or by leveraging differences in water molecule diffusion between cells and the extracellular compartments (see the “Noninvasive imaging and measuring methods of the human brain” section and Fig. 6),

**Table 1.** Brain ECS measurement methods

Methodology	Tracer-based methods with probes			EM/nanoscopy			DWI		
Modality	RTI-TMA^+^ [16]	IOI [25]	TB-MRI [26]	FS-EM/HPF-EM [17]	SUSHI [209]	CNTs [32]	DTI-ALPS [51]	NODDI/VERDICT [47,49]	MDI [50]
Experimental setting	Ex vivo*/* in vivo	Ex vivo*/*in vivo	In vivo	In vitro	Ex vivo	Ex vivo	In vivo	In vivo	In vivo
Probe/stain	TMA^+^	Dextran	Gd-DO3A-EA-FITC/Gd-DTPA	Osmium tetroxidee	Atto-514	SWCNTs	Endogenous ^1^H	Endogenous ^1^H	Endogenous ^1^H
Spatial scale	<200 μm in depth	<400 μm in depth	Whole brain	Whole brain	<200 μm in depth	<200 μm in depth	Whole brain	Whole brain	Whole brain
Dimension	1D	2D	3D	2D/3D	2D	2D	3D	3D	3D
Anisotropy/isotropy	Isotropic	Isotropic	Anisotropic	Anisotropic	Anisotropic	Anisotropic	Anisotropic	Anisotropic	Anisotropic
Parameters	*D*, *α*, *λ*	*D*, *α*, *λ*	*D*, *α*, *λ*, *t*_1/2_	*α*	*α*	*D*, *α*	ALPS index	ICVF, FISO	*D*, *α*, FA

Gd-DO3A-EA-FITC, gadolinium(III)[4,7-bis-carboxymethyl-10-(2-fluoresceinthioureaethyl)-1,4,7,10-tetraaza-cyclododec-1-yl]-acetic acid; Gd-DTPA, gadolinium(III) diethylenetriaminepentaacetate; *D*, diffusion coefficient; *α*, volume fraction; *λ*, tortuosity; *t*_1/2_, half-life; ALPS index; ICVF, intraneurite volume fraction; FISO, fraction of isotropy.

### Measurement methods based on solving the inverse problem of the diffusion equation

ECS measurement methods based on solving the inverse problem of the diffusion equation share a common technical feature that they require introducing specifically distributed tracers into the ECS and detecting their spatiotemporal concentration distribution within this space [16,25]. Subsequently, the physical parameters of the ECS (i.e., *D*, *α*, and *λ*) are calculated by solving the inverse problem of the advection–diffusion equation [23,25]. Substance transport within the porous medium of the ECS can be described by the general advection–diffusion equation [27]:

$$\frac{\partial C}{\partial t}=\nabla \cdot \left(D\nabla C\right)-v\cdot \nabla C-\mathrm{kC}+\frac{Q}{\alpha }$$ (1)

where *C* denotes the tracer concentration at time *t* and position (*x*, *y*, *z*), *D* represents the diffusion tensor that characterizes anisotropic diffusion properties in the porous medium, *v* is the velocity vector field with components in all spatial directions, *k* is the clearance rate coefficient associated with the tracer’s in vivo metabolism, *Q* is the source term introduced into the ECS, and *α* represents the volume fraction of the ECS. Tortuosity λ is typically introduced to characterize the hindrance posed by the porous medium to diffusion. The relationship between the effective diffusion coefficient $D^{*}$ and the diffusion coefficient in a free solution *D* is defined as $D^{*}=\frac{D}{\lambda^{2}}$. Constrained by limitations in imaging dimensions and resolution, existing ECS measurement and calculation techniques usually apply simplifications to the physical models and boundary conditions to achieve practical engineering solutions.

#### Measurement of ECS diffusion with 1- or 2-dimensional inverse problem solution to the diffusion equation

##### Real-time iontophoresis–tetramethylammonium

Real-time iontophoresis–tetramethylammonium (RTI-TMA^+^) is a classic approach, which utilizes a microelectrode to release TMA^+^ as a point source into the tissue, subsequently recording precise concentration changes at a preset distance [28,29]. Mathematically, this method neglects the advection term and, based on the assumption of medium isotropy, simplifies Eq. (1) from a Cartesian coordinate system to a spherically symmetric radial diffusion equation for solving the inverse problem:

$$\frac{\partial C}{\partial t}=D^{*}\frac{1}{r^{2}}\frac{\partial }{\mathrm{\partial r}}\left(r^{2}\frac{\partial C}{\mathrm{\partial r}}\right)+\frac{Q}{\alpha }-\mathrm{kC}$$ (2)

where r denotes the radial distance from the microelectrode tip. Early methodologies often simplified transport dynamics by neglecting k and calculating α directly from steady-state concentration ratios between brain and agar [16]. Conversely, subsequent methods commonly use multiparameter nonlinear fitting to obtain more accurate values, yielding an α value of approximately 0.2 for normal brain parenchyma [29]. In addition, for oriented tissues such as white matter, anisotropic models expand the effective diffusion coefficient into a tensor with independent tortuosity components along principal axes [30].

##### Integrative optical imaging

Integrative optical imaging (IOI) uses pressure pulses to introduce fluorescent tracer (e.g., dextrans), establishing an approximate instantaneous point source [25,31]. Leveraging the large depth-of-field characteristics of epifluorescence microscopy, the method performs an optical integration of the 3-dimensional (3D) diffusion cloud along the optical axis. Mathematically, the projection of the 3D probe distribution onto the 2D imaging plane follows a Gaussian distribution. The fluorescence intensity $I\left(r\right)$ at radial distance r from the source is described as follows:

$$I\left(r\right)=E\;\exp\left[-{\left(\frac{r}{\gamma }\right)}^{2}\right],\;\gamma^{2}=4D^{*}\left(t+t_{0}\right)$$ (3)

where *I*(*r*) represents the fluorescence intensity at a radial distance *r* from the injection site; E denotes the amplitude coefficient, which integrates the out-of-focus point spread function, the initial concentration of the probe, and the calibration factor of the optical system; and $t_{0}$ is the time offset for the onset of diffusion. By fitting the temporal evolution of γ across multiple image frames, $D^{*}$ can be extracted, allowing for the subsequent calculation of tortuosity. Combined with restricted diffusion theory, this method estimates the effective width of the ECS in normal brain tissue to be approximately 38 to 64 nm, thereby overcoming the underestimation inherent in traditional EM caused by tissue shrinkage. However, the tissue homogeneity assumption fails at structural boundaries [30], and the rigid sphere model may underestimate tortuosity in heterogeneous tissue regions as revealed by nanoscale tracking studies [32], limiting accuracy when applied to complex brain tissue such as complex fiber architectures or perivascular spaces (PVSs).

#### Measurement of ECS diffusion using 3D inverse problem solutions to the diffusion equation

To address the depth limits of optical imaging and resolve the anisotropic characteristics of the ECS, Han et al. [26] developed the tracer-based magnetic resonance imaging (TB-MRI) (Fig. 3). Paramagnetic or photomagnetic probes were introduced into the brain ECS to trace and lighten the water molecules within the space. The spin–lattice relaxation time of hydrogen nuclei in water molecules was shortened, which presented as a high signal on MRI. The average width between neural cells is 20 to 130 nm, and the effective distance for the probe to lighten the hydrogen nuclei in water molecules ranges from 2.41 to 2.50 Å. The enhancement on MRI decreased over time because of the water diffusion and clearance process of the probes. This process was dynamically recorded on a series of magnetic resonance (MR) images [8,26]. In terms of mathematical modeling, ECS diffusion measurement is realized by solving the inverse problem of the following 3D anisotropic diffusion equation:

$$\frac{\mathrm{\partial C}}{\partial t}=D_{x}\frac{\partial^{2}C}{\partial x^{2}}+D_{y}\frac{\partial^{2}C}{\partial y^{2}}+D_{z}\frac{\partial^{2}C}{\partial z^{2}}-\mathrm{kC}+\frac{Q}{\alpha }$$ (4)

This method enables voxel-level quantification of whole-brain ECS parameters (*D*, *α*). On the basis of TB-MRI technology, the research team revealed the partitioned drainage characteristics of brain ISF for the first time, correcting the traditional view regarding the high connectivity of the ECS across the entire brain [33].

#### Measurement of ECS diffusion using inverse problem solutions to the advection–diffusion equation

Previous ECS quantification methods generally adopted a simplified approach by omitting the advection term of the advection–diffusion equation, thereby yielding only diffusion-related parameters. However, the presence of convective transport in the ECS is a historically controversial topic [28,34]. While Ray et al. [34] utilized simulations to address combined diffusion and flow, such methods are constrained by high computational costs, and a lack of practical applicability [24] addresses the inherent complexity of this inverse problem, Xie et al. [35] sought to address the inverse problem derived from the following advection–diffusion equation by utilizing a physics-informed neural network (PINN) and the 3D whole-brain ECS data acquired with the TB-MRI:

$$\frac{\partial C}{\partial t}=\nabla \cdot \left(D\nabla C\right)-v\cdot \nabla C$$ (5)

This approach establishes a technical pathway that allows for the simultaneous quantification of both diffusion and advection parameters within the ECS (Fig. 4) [35]. To further enhance the mathematical stability and uniqueness of the solution, topological constraints from high-precision EM and diffusion priors from multicompartment decoupling imaging (MDI) could be integrated to effectively constrain the solution space. This strategy offers a promising pathway for resolving long-standing controversies regarding ECS transport mechanisms.

### EM and other nanoscopy methods

#### Electron microscopy

In 2023, Huang et al. [17] established an in situ imaging system that utilized focused ion beam scanning EM for high-resolution 3D EM. This approach achieved the first 3D visualization of the natural ECS structure in wild-type rat hippocampal CA1 tissue. The study quantified the ECS volume fraction to be approximately 6.21%, significantly lower than the results derived from mathematical modeling techniques [28]. Research in other brain regions (e.g., thalamus [Tha]) has shown that the volume fraction can reach up to 15% in certain areas. By incorporating contrastive learning and shape-aware modules, Yang et al. [18] proposed a dedicated segmentation network, ECS-Net, which addressed the challenges of interclass feature imbalance and fine structural recognition, achieving high-precision automated segmentation and reconstruction of the ECS (Fig. 5). With AI-aided high-precision reconstruction of the ECS, cells, and vasculature, it is now possible to construct cross-scale simulation models that connect microstructural and macroscopic functions, allowing for quantitative analysis of their interactions under various physiological and pathological conditions [36–38].

#### Tracer-based optical imaging

Tracer-based optical imaging methods investigate the diffusion properties and microstructural characteristics of the brain ECS using exogenous fluorescent probes. Most studies are performed on brain slices, allowing visualization of probe dynamic distribution within a depth range of 200 to 400 μm [39]. Recent advances have integrated nanoparticle-based probes and super-resolution imaging strategies. Among these, single-walled carbon nanotubes (SWCNTs), with low phototoxicity, high photostability, and minimal immunoreactivity, are used for long-term single-particle tracking in brain tissue [40]. Analyzing the anisotropic Brownian trajectories of SWCNTs in the ECS has elucidated the nanoscale heterogeneity of the ECS [32].

Stimulated emission depletion (STED) microscopy achieves ultrahigh spatial resolution beyond the diffraction limit via phase modulation to form a “donut”-shaped spot with zero central intensity, enhancing the visualization of subcellular nanostructures. It reveals ECS changes during cerebral ischemia and neuroinflammation, as well as their impacts on drug delivery and metabolic waste clearance [41]. By injecting fluorescent dyes into the ECS and combining with STED imaging, SUSHI uses negative contrast labeling of the ECS to quantitatively measure local ECS width, connectivity, and dynamic structural remodeling, while resolving spatial proximity between the ECS and adjacent neuronal/vascular structures within the same field of view [19,41,42].

Tracer-based optical imaging methods offer distinct advantages in resolving local ECS architecture and nanoscale diffusion behaviors, rendering them a valuable option for investigating spatial heterogeneity at the micro- and subcellular levels. However, these methods are hampered by several fundamental limitations: First, both the probes and excitation light must exhibit high biocompatibility to avoid perturbing normal tissue physiology or inducing phototoxicity. Second, most fluorescent probes are not ECS-specific, and their nonspecific cellular uptake or retrograde passage across the BBB can interfere with imaging results and quantitative analysis. In addition, inherent physical limitations, including limited imaging field of view, restricted tissue penetration, and challenges in cross-scale synchronization. All these require continuous technological advancements to overcome, which further confines their utility primarily to brain slices or localized tissue regions. As research focus shifts toward understanding global ECS transport functions and system-level regulation in the intact living brain, there is an increasing demand for techniques with whole-brain in vivo imaging capabilities. In this context, noninvasive MRI has emerged as a key approach for studying the human brain ECS.

### Noninvasive imaging and measuring methods of the human brain

Recent studies have demonstrated that modulating substance transport within the ECS represents a viable therapeutic strategy for neurological disorders [43,44]. These findings have further stimulated enthusiasm for imaging the human brain ECS and quantitatively measuring substance transport within it. Single-photon emission computed tomography has been used to visualize amyloid-β (Aβ) protein deposition within the ECS [45], enabling semiquantitative parameter calculation via isotope labeling; however, its utility is constrained by limited spatial resolution and specificity. In contrast, MRI exhibits considerable advantages in ECS structural analysis owing to its noninvasiveness, absence of ionizing radiation, and superior resolution compared to nuclear imaging, establishing diffusion MRI as the preferred method for ECS quantification in the human brain.

Diffusion-weighted imaging (DWI), which characterizes MR signal attenuation induced by water molecule diffusion within tissues based on the Stejskal–Tanner equation, serves as the core technology for the noninvasive detection of diffusion properties. Currently, several advanced DWI-based analytical models are utilized for assessing the ECS characteristics, including diffusion tensor imaging analysis along the PVS (DTI-ALPS) [46], neurite orientation dispersion and density imaging (NODDI) [47], vascular, extracellular, and restricted diffusion for cytometry in tumors (VERDICT) [48,49], and MDI [35,50].

DTI-ALPS evaluates PVS function via direction-dependent water diffusion near the lateral ventricles [46]. The ALPS index is defined as follows:

$$\text{ALPS}=\frac{\text{Mean}\left(D_{x}^{\text{proj}},\;D_{x}^{\text{assoc}}\right)}{\text{Mean}\left(D_{y}^{\text{proj}},\;D_{z}^{\text{assoc}}\right)}$$ (6)

Here, $D_{x}^{\text{proj}}$ and $D_{x}^{\text{assoc}}$ denote the diffusivities along the left–right x axis in the projection and association fiber regions of interest (ROIs), respectively; $D_{y}^{\text{proj}}$ is the diffusivity along the anterior–posterior y axis in the projection fiber ROI; and $D_{z}^{\text{assoc}}$ represents the diffusivity along the superior–inferior z axis in the association fiber ROI. Since the PVS runs along the x axis, a larger ALPS value indicates relatively higher PVS diffusivity. This approach facilitates monitoring diseases such as AD [51], although fiber microstructural asymmetry may confound results [52].

NODDI characterizes the microstructure complexity of dendrites and axons by modeling the normalized diffusion MR signal as a 3-compartment mixture:

Sb,gS0=1−fisoficEicb,g+1−ficEecb,g+fisoEisob (7)

Here, $S\left(b,,,\;,g\right)$ is the signal measured with diffusion weighting b along gradient g relative to the baseline $S_{0}$; the estimated parameters $f_{\mathrm{iso}}$ and $f_{\mathrm{ic}}$ correspond to the volume fractions of CSF and neurite density, respectively; $E_{\mathrm{ic}}$ $E_{\mathrm{ec}}$, and $E_{\mathrm{iso}}$ are the modeled signal attenuations from the intracellular, extracellular, and isotropic compartments, respectively [47]. Recently, VERDICT adapted this framework by replacing CSF with a vascular compartment and assuming isotropic water diffusion within the ECS [48,49].

Evaluating ECS-based neuromodulation requires noninvasive and quantitative measurements of both cellular and ECS diffusion dynamics. Li et al. [50] developed the MDI technique [35]. MDI uses diffusion-weighted MRI sequences to acquire image signals that encapsulate molecular diffusion information across multiple compartments, performs multicompartment modeling of diffusion signal attenuation [Eq. (8)], and applies constrained nonlinear optimization to quantitatively calculate and characterize the water diffusion properties of the cellular, extracellular, and vascular compartments as

$$\begin{array}{c} \begin{array}{c} S\left(b,\;g\right)=S_{0}\left[\alpha_{\text{cell}}e^{-bg^{T}D_{\text{cell}}g}+\alpha_{\mathrm{ecs}}e^{-bg^{T}D_{\mathrm{ecs}}g}+\alpha_{\mathrm{vas}}e^{-bg^{T}D_{\mathrm{vas}}g}\right] \end{array}\\ s.t.,\alpha_{\text{cell}}+\alpha_{\mathrm{ecs}}+\alpha_{\mathrm{vas}}=1 \end{array}$$ (8)

Here, $S\left(b,\;g\right)$ and $S_{0}$ denote the diffusion-weighted and baseline signals; $\alpha_{\text{cell}}$, $\alpha_{\mathrm{ecs}}$, $\alpha_{\mathrm{vas}}$ and $D_{\text{cell}}$, $D_{\mathrm{ecs}}$, $D_{\mathrm{ecs}}$ represent the voxel-wise volume fractions and diffusion tensors for the cellular, ECS, and vascular compartments, respectively. The resulting voxel-level parameters enable quantitative mapping of the 3 compartments across multiple spatial scales: nanometer-scale ECS, micrometer-scale neurons/neural cells, and millimeter-to-micrometer-scale microvasculature (Fig. 6).

As a tool for simultaneous 3-compartment information acquisition and quantification, MDI offers a novel pathway for elucidating brain disease mechanisms and identifying potential therapeutic targets. Recent advances in high-pressure freezing EM enable direct visualization of native ECS structures, while AI-assisted image processing techniques provide robust methodological support for the potential modification and validation of MDI. Large-scale multicenter clinical trials using MDI are essential to integrate the ECS concept into the current framework of novel drug and medical device development [53].

Table 1 summarizes the main characteristics of ECS imaging and measurement methods, illustrating the evolution of this field from tracer-based, ex vivo approaches toward label-free, in vivo, and clinically applicable techniques. Key technological milestones over the past decade, such as AI-assisted cryo-EM reconstruction, super-resolution live imaging, and quantitative clinical MRI, have progressively overcome the core technical bottlenecks that previously hindered the integration of the ECS into neuroscience research and therapeutic development. By virtue of these tools, the ECS should no longer be regarded as a passive space but must be systematically studied as a structurally and functionally critical domain. This series of technical advances paves the way for integrating the ECS into central CNS therapeutic paradigms.

## Latest Discoveries of the Brain ECS Influencing Brain Disease Treatment Strategies

With the advancements in ECS measurement and imaging technologies, particularly breakthroughs in deep brain detection capabilities, a series of compelling scientific discoveries have emerged in the field of the brain ECS. Among these, 3 findings hold profound implications for the innovation of therapeutic strategies for brain disorders: the identification of the brain ECS parcellation system, the visualization of the native architecture of the ECS, and the active modulation of substance transport within the brain ECS. This section first introduces the parcellation of the brain ECS (see the “The discovery of the brain ECS parcellation” section), subsequently elaborates on the interactive cross-talk between the ECS and its adjacent structural compartments (see the “Substance transportation in the brain ECS” section), and finally discusses recently developed technical approaches enabling the active modulation of substance transport in the ECS (see the “Modulation of substance transport within the brain ECS” section).

### The discovery of the brain ECS parcellation

**Fig. 7.**
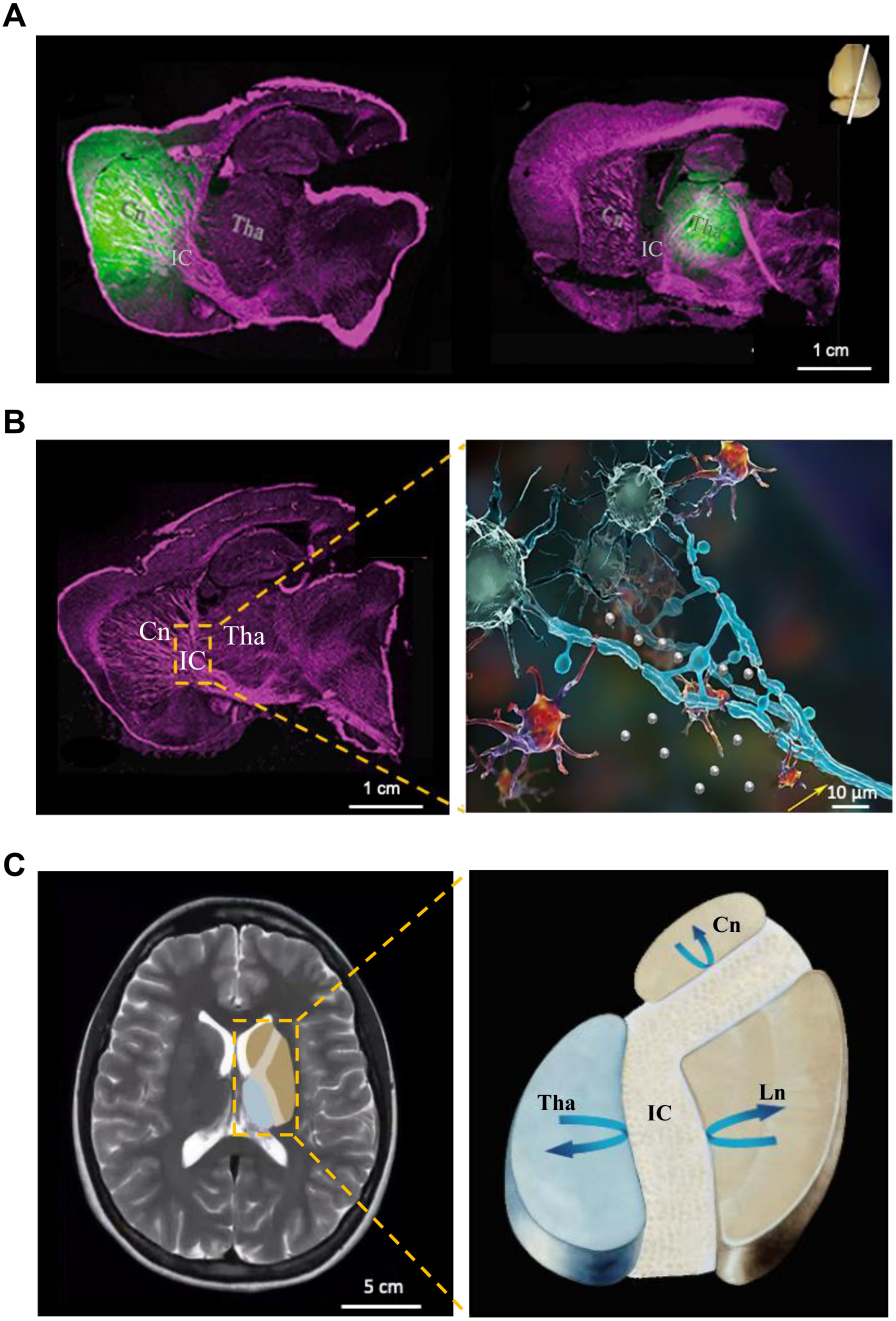
ECS parcellation and substance transport routes in the brain ECS. (A) Substances within the ECS of the Cn are unable to translocate to the ECS of the Tha and vice versa, despite the anatomical adjacency of these 2 brain regions. (B) The structural barrier that restricts interregional translocation from the Cn to the Tha is identified as mature myelinated fiber tracts in the adult rat brain. IC, internal capsule. (C) Schematic illustration of ECS parcellation in the human basal ganglia. Arrows denote that substances in distinct basal ganglia nuclei are impeded from traversing the compact internal capsule, thereby sustaining the microenvironmental homeostasis essential for neuronal activity within individual nuclei. Ln, lentiform nucleus.

As the fundamental framework for deciphering the structure–function relationship in the brain, 2 classical parcellation systems have dominated the field of neuroscience: the Brodmann system and the vascular territory system. The Brodmann system is delineated on the basis of the cytoarchitectural features of the cerebral cortex, whereas the vascular territory system is defined by patterns of arterial supply and venous drainage [54–56]. However, neither of these classical paradigms accounts for the spatial transport pathways or diffusion profiles within the ECS. Utilizing TB-MRI, Han et al. demonstrated that tracers injected into the caudate nucleus (Cn) and Tha of adult rat brains were strictly confined to their respective drainage territories, with no cross-territory communication detected. Specifically, tracers from the Cn drained primarily along myelinated fiber tracts toward the ipsilateral cortex, with a small fraction entering the fourth ventricle via the ependyma; in contrast, tracer diffusion into the thalamic ECS was completely blocked by dense myelinated barriers. These findings were corroborated using optical imaging (Fig. 7), indicating that the Tha, Cn, and internal capsule are not only structurally discrete but also exhibit distinct ISF drainage properties [33,57].

On the basis of these observations, subsequent investigations have confirmed that this ECS barrier is not innate but rather develops postnatally, reaching maturity in rats at approximately postnatal day 40. Prior to the full maturation of this ECS barrier, immature myelin results in the absence of barrier function, permitting unimpeded cross-regional diffusion of neurotransmitters (e.g., dopamine) from the Cn to the Tha [58,59]. As brain structures undergo progressive maturation, the densification of myelin lamellae (with thickness increasing from 0.14 to 0.24 μm) establishes a functional barrier that fully blocks this passive diffusion. This spatial segregation sustains distinct ionic and neurochemical homeostasis, thereby optimizing the function of the cortex–striatum–Tha circuit and enhancing cognitive performance (Fig. 7) [59].

In contrast, aging is characterized by myelin degeneration, attenuated arterial pulsatility, and cerebrovascular dysregulation. All the above factors compromise ECS barrier integrity, reduce ISF drainage efficiency, and impair waste clearance capacity. Studies have demonstrated that aged mice (18 to 20 months old) exhibit significantly lower ISF clearance rates compared with young controls (2 to 3 months old) (Table 2) [60,61]. Ultimately, impaired waste clearance perturbs the neural microenvironment and accelerates cognitive decline; notably, this mechanism may underlie the heterogeneity of age-related cognitive impairment.

**Table 2.** Alterations of the brain ECS across different age stages

Region	Method/probe	Species	Age	*α*	*λ*	D* × 10^−4^/(mm^2^·s^−1^)	Refs.
Cortex	RTI-TMA^+^/ TMA^+^	Wistar rats	Juvenile	0.260 ± 0.020	1.610 ± 0.025		[210]
		Wistar rats	Adult	0.210 ± 0.010	1.650 ± 0.030		[211]
		Wistar rats	Aged	0.180 ± 0.010	1.590 ± 0.020		[212]
Striatum	TB-MRI/Gd–DTPA	SD rats	Juvenile (P20)	0.180 ± 0.030	1.610 ± 0.050	3.90 ± 0.460	[59]
		SD rats	Juvenile (P40)	0.174 ± 0.040	1.710 ± 0.060	3.62 ± 0.260	[213]
		SD rats	Adult	0.173 ± 0.001	1.760 ± 0.020	3.43 ± 0.950	[213]
Hippocampus	RTI-TMA^+^/TMA^+^	Wistar rats	Juvenile (P5)	0.410 ± 0.010	1.390 ± 0.010		[214]
		Wistar rats	Adult	0.214 ± 0.004	1.622 ± 0.006		[215]
		Wistar rats	Aged	0.186 ± 0.004	1.599 ± 0.010		[216]

SD, Sprague–Dawley.

### Substance transportation in the brain ECS

Building on the parcellation and barrier properties of the brain ECS discussed above, substance transport within the brain ECS is a complex process regulated by multiple factors (Table 3). Diverse physiological alterations, including the functional activities of adjacent neurons, glial cells, and vascular structures, can modulate the spatial configuration of the ECS as well as substance transport within it. Conversely, alterations in ECS properties may reciprocally influence the states of surrounding neurons and neural networks.

**Table 3.** Factors influencing substance transport in the normal brain ECS

Adjacent structure or physiological factors	Species	Biological mechanism	Effect on ECS transport	Refs.
Neuron	Rat	The chemical changes following neuronal excitation, including released neurotransmitter, ion exchange, etc.	Diffusion and ISF drainage are down-regulated following neuronal excitation under anesthesia.	[40,60,63,64,70,71,217]
Oligodendrocyte	Rat/mouse	Acts as ECS barrier and transport conduit	Local or extensive demyelination causes disturbance of transportation in the brain ECS	[58,59]
Astrocyte	Rat	Regulates the transport in the ECS via AQP4	ISF drainage function is impaired in AQP4 gene knockout models	[73]
Cerebrovascular vessel	Mouse/rat/ human	Mechanical force effect	Arterial pulsation facilitates CSF inflow, accelerating the rate of CSF–ISF exchange and substance transport in the ECS	[43,74,75,77,95]
CSF	Mouse	Substances in the superficial cortex ECS exchanges with the CSF.	The subarachnoid space serves as a relay station for substance transport within the ECS	[78–80]
Lymphatic circulation	Dog/mouse/rat/rabbit/cat	Downstream of the ECS transport to enter the systemic circulation	Ligation of cervical lymph nodes slows ISF flow; dural lymphatic epithelium is the intermediate link	[140,141,218]
Respiration	Human	Intrathoracic pressure affects intracranial venous pressure via the jugular vein	Drive CSF into the PVS and back to the venous system	[76,77]

Neuronal excitation induces alterations in diffusion within the brain ECS, characterized by a prolonged tracer half-life [40,62]. In a rat model of electrical pain stimulation under combined anesthesia, neuronal excitation was shown to induce a significant reduction in diffusion rate and ISF drainage. Using mass spectrometry imaging and gene knockout technology, the alterations in brain ECS transportation were verified to be primarily attributable to biochemical changes rather than morphological modifications of the ECS [62].

Notably, inhalational and intravenous anesthetics with distinct anesthetic mechanisms exert differential effects on ECS substance transport. Compared with isoflurane, dexmedetomidine- and sodium-pentobarbital-induced anesthesia results in a significant reduction in ECS tracer half-life; this difference is associated with cerebral neurotransmitter levels (e.g., norepinephrine) [63]. This finding provides theoretical underpinnings for the application of dexmedetomidine combined with low-dose isoflurane in balanced anesthesia, thereby mitigating postoperative delirium. In addition, Lilius et al. [64] demonstrated that dexmedetomidine enhances slow-wave electroencephalogram activity and accelerates CSF–ISF exchange in the superficial cerebral cortex.

Furthermore, neuronal excitation induces both increased electrical discharge frequency and reduced ECS volume, which collectively promote the extracellular accumulation of neuroactive substances. This, in turn, further amplifies neuronal excitability, thereby forming a positive feedback loop that exacerbates perturbations in the local neural microenvironment [28,65,66]. Subsequently, during the refractory period following neuronal excitation, enhanced ion transport facilitates the reestablishment of ionic concentration gradient equilibrium across the cell membrane [67]; meanwhile, the reuptake or enzymatic degradation of extracellular neurotransmitters promotes the resolution of astrocytic edema and the restoration of ECS structural integrity [68,69]. This dynamic regulatory interplay has been further validated via ECS–neuron interaction simulations [28].

Notably, studies focusing on the ECS during sleep further validate that neuronal excitation suppresses molecular transport within the ECS. Reduced levels of neuronal excitability shorten the tracer half-life and accelerate substance diffusion in the ECS [63,70]. Mechanistic investigations reveal that during sleep, slow-wave oscillations elicited by synchronous neuronal action potentials generate rhythmic ionic waves in the cerebral ISF, expediting CSF–ISF exchange and thereby enhancing metabolic waste clearance [71]. Similarly, deep meditation induces slow-wave cerebral electrical activity, reduces norepinephrine secretion, and recapitulates a sleep-like cerebral waste clearance profile [72].

As the major glial cell populations, both oligodendrocytes and astrocytes are intimately involved in substance transport within the ECS. Oligodendrocyte-derived myelin sheaths serve as either ISF drainage conduits or barriers, whereas astrocyte-associated aquaporin-4 (AQP4) modulates ECS substance transport. Specifically, ISF drainage of Cn in the deep brain regions is significantly impaired in AQP4 knockout mice, suggesting that AQP4 regulates the flow dynamics of ISF by modulating water movement between the ECS and astrocytic endfeet [73].

Beyond glial cells and neuronal activity, vascular pulsation represents a key determinant of CSF–ISF exchange. Studies have demonstrated that unilateral internal carotid artery ligation, via eliminating arterial pulsation, markedly reduces CSF–ISF exchange efficiency, whereas adrenergic agonists enhance this exchange efficiency by amplifying arterial pulsatility [74]. In spontaneously hypertensive rats, accelerated fluorescent tracer diffusion in the hippocampal ECS may correlate with elevated pulse pressure and altered pulsatility profiles [75] dynamics induced by respiration also contribute to the regulation of CSF–ISF drainage [76]. During inspiration, negative thoracic pressure propagates intracranially via the jugular venous system, dilating the perivenous space and reducing local pressure, thereby driving CSF–ISF influx along the pressure gradient. Conversely, expiration generates positive intrathoracic pressure, which elevates intracranial venous pressure and compresses the perivenous space. This respiratory “squeezing effect” actively facilitates CSF–ISF reflux into the venous system [77].

With respect to the clearance pathways of ECS molecules in the superficial cortex, these molecules traverse the leptomeningeal–glial membrane into the subarachnoid space, undergo thorough mixing with CSF, and subsequently enter the peripheral circulation via venous and lymphatic routes [78,79]. CSF–ISF efflux occurs through 2 distinct pathways: approximately 70% to 80% is cleared via arachnoid granulations into the dural sinuses and then into the internal jugular vein; the remaining fraction drains through peribridging venous arachnoid cuff exits into dural lymphatic vessels, ultimately reaching the deep cervical lymph nodes [80].

### Modulation of substance transport within the brain ECS

Substance transport within the ECS can be actively modulated through physical or pharmacological interventions (Table 4). Targeted and precise regulation of the ECS affords promising novel therapeutic strategies for the treatment of brain disorders.

**Table 4.** Neuromodulation methods and their effects on the brain ECS

Types of modulation	Noninvasive/invasive	Modulation technical parameters	Brain disease models and species	Therapeutic target	Modulation effects	Refs.
Light stimulation	Minimally invasive	1,050 nm	Intraventricular hemorrhage mice	Increase the production of NO	Facilitate ISF drainage	[95]
		1,267 nm				
		808 nm	AD mice	Enhance meningeal lymphatic endothelial cells (mLECs)’ mitochondrial function	Facilitate ISF drainage	[84]
		630 nm	AD mice/healthy mice	Promote Aβ disassembly	Facilitate ISF drainage, increase local diffusion rate, and decrease ECS tortuosity	[219, 220]
		LED (40 Hz)	AD mice	Increase arterial pulsatility and AQP4 polarization	Promote the influx of CSF	[221]
		LED (60 Hz)	Healthy mice	Release of MMPs	Affecting the ECM components	[222]
Ultrasound stimulation	Noninvasive	MRI-guided FUS	Gliomas and melanomas mice	Increase the permeability of the BBB	Increase ISF flow velocity	[89]
		FUS combined with microbubables (MBs)	Healthy mice	Ca^2+^ influx induces c-Fos up-regulation	Enhance ECS substance transport	[91]
Synchronous pulsation	Invasive	Unilateral ligation of the internal carotid artery	C57BL/6 mice	Astroglial water transport via the AQP4	Decrease CSF flow	
		–	Spontaneously hypertensive rats (SHR) and normotensive Wistar Kyoto (WKY) rats	Water content within ECS and changes in Na^+^/K^+^ ratios	Increase ISF flow	[75]
		EAI	Adult male SD rats	The arterial pulsation accelerates the exchange between CSF and ISF	Increased ISF drainage and ECS volume fraction reduced ECS tortuosity and increased local diffusion rate	[95,43]
		Forced breathing	Healthy subjects	Inspiratory thoracic pressure reduction	Enhanced brain ISF drainage	[223]
		Cardiac, respiratory, and very-low-frequency and low-frequency waves	Healthy subjects	Cardiac pulsations propel water through aquaporin channels and support solute transport	Facilitate CSF flow	[77]
Electrical stimulation	Invasive	tDCS	Healthy mice	Through astrocytic IP_3_/Ca^2+^ signaling, modify the delta wave	Increased solute diffusion in ECS	[85,86]
		Electroacupuncture	AD mice	–	Increase ECS clearance	[87]
		DBS	PD rats	Improve AQP4 polarity	Increased ISF drainage and ECS volume fraction, reduced ECS tortuosity, and increased local diffusion rate	[164]
TMS	Noninvasive	Continuous theta burst stimulation	C57BL/6J mice	Improve AQP4 polarity	Increase ECS substance transport	[88,224,225]
		Other rTMS	C57BL/6J mice	Activate GABA	Improve ECS waste clearance	[225]

LED, light-emitting diode; GABA, γ-aminobutyric acid.

Photobiomodulation therapy (PBMT) uses nonthermal red light (RL; 630 to 800 nm) or near-infrared (NIR; 780 to 1,100 nm) light to promote tissue repair and regeneration [81]. PBMT at different wavelengths exerts differential effects: 630-nm RL accelerates ISF-CSF circulation, restores ECS structural integrity, reduces Aβ aggregation, and inhibits neuronal apoptosis. Mechanistically, PBMT with 630-nm enhances nitric oxide (NO) generation in meningeal lymphatic endothelial cells, thereby modulating vascular dilation and accelerating both brain ISF drainage and waste clearance. When combined with nanotherapeutics capable of penetrating ECS pores (38 to 64 nm), such as 30-nm encapsulated coenzyme Q10, 630-nm irradiation synergistically reduces formaldehyde accumulation and Aβ deposition in amyloid precursor protein/presenilin 1 mice, ameliorating memory deficits; PBMT with 808-nm laser therapy restores mitochondrial metabolism in meningeal lymphatic endothelial cells, modulates lymphatic contractility, and enhances ISF clearance to mitigate AD-related pathology; similarly, pulsed 1,070-nm NIR irradiation (10 Hz) activates microglia to enhance Aβ degradation, restores ISF drainage, and mitigates cognitive impairment in AD models. Cumulative evidence demonstrates that PBMT mitigates neurodegeneration associated with conditions such as stroke, traumatic brain injury, Parkinson’s disease (PD), and AD [82–84].

In addition to PBMT, electrical and magnetic stimulations are also effective strategies for modulating substance transport in the ECS. Deep brain stimulation (DBS) alleviates PD-related pathology in preclinical models by reconfiguring the local neurotransmitter microenvironment, accelerating ECS molecular diffusion and drainage, and restoring neurotransmitter homeostasis. Transcranial direct current stimulation (tDCS) is a noninvasive technique delivering low-intensity currents (<2 mA) to targeted brain regions, which can enhance diffusion and BBB permeability, thereby regulating ECS function [85]. Mechanistically, tDCS promotes ISF drainage via astrocytic inositol 1,4,5-trisphosphate (IP_3_)/Ca^2+^ signaling and modulates δ-wave activity associated with cerebral waste clearance [85,86]. Similarly, electroacupuncture enhances perivascular glymphatic influx, improves AQP4 polarization, and reduces Aβ deposition in AD models [87]. Repetitive transcranial magnetic stimulation (rTMS) also facilitates the clearance of neuropathological proteins (e.g., Aβ) from the ECS by enhancing AQP4 polarization [88].

Transcranial ultrasound stimulation (TUS) enables safe and efficient targeting of specific brain regions via continuous or pulsed acoustic energy. Focused ultrasound (FUS) accelerates fluorescent tracer transport in arteriole-dominant PVS and enhances tracer penetration into the ECS, thereby modulating the BBB to improve the delivery, distribution, and uptake of therapeutic agents in the brain. As a precise deep brain neuromodulation strategy, FUS holds considerable promise for the treatment of AD and glioblastoma [89–92]. Notably, MRI-guided FUS achieves transient, localized BBB opening to accelerate transportation in the ECS and perivascular influx, without compromising long-term BBB integrity or inducing parenchymal damage [93,94]. Collectively, the noninvasive and low-intensity characteristics of TUS indicate its strong potential for rapid clinical translation.

Surgical intervention is an alternative strategy for regulating ECS substance transport. Han et al. developed an epidural artery implantation (EAI) technique that synchronizes with cerebral pulsations, thereby enhancing substance transport in the downstream drainage regions of the deep brain ECS. Even in the absence of pharmacological neuroprotectants, EAI accelerates ISF drainage in both the superficial cortex and deep brain regions, promotes the clearance of proinflammatory cytokines, alleviates inflammatory responses, and exerts neuroprotective effects in the context of ischemic stroke [43,95].

## Therapeutic Strategies and Paradigm Innovation with Brain ECS Knowledge

Traditional therapeutic strategies for brain diseases have historically overlooked the brain ECS, which fails to fully elucidate the pathogenesis of brain disorders and thereafter is hard to identify the most optimal treatment protocols. A systematic paradigm shift from the traditional “cell–vasculature” framework to a “cell–ECS–vasculature” framework will provide a structurally complete mechanism for the onset, progression, and outcome of brain diseases. Furthermore, this evolution will not only facilitate the identification of precise therapeutic targets and optimize neuromodulation techniques but also provide an efficient drug delivery route distinct from that of the traditional vascular delivery pathway [11]. The following sections illustrate the value of integrating the ECS into mechanistic research and evaluation, focusing on AD and ischemic stroke. Finally, the technical prerequisites for regulatory paradigm innovation are discussed.

### Incorporating pathological ECS changes into research framework of brain diseases

AD is the leading cause of cognitive impairment worldwide. The typical pathological changes of AD are senile plaques (SPs) formed by Aβ protein deposits in the ECS and neurofibrillary tangles (NFTs) formed by hyperphosphorylated tau protein within the neuronal cytoplasm [96]. For a long time, drug development for brain diseases has primarily focused on molecular-level intervention targets [97], mainly targeting neurotransmitter dysregulation in affected areas and Aβ deposition in the ECS [98]. However, the failure of such drug development has prompted the industry to explore novel therapeutic strategies [99,100]. As the microenvironment sustaining neuron survival and the new potential pathway for drug or physical treatment [26], the ECS provides a totally new perspective for overcoming the bottlenecks in AD treatment [101].

**Fig. 8.**
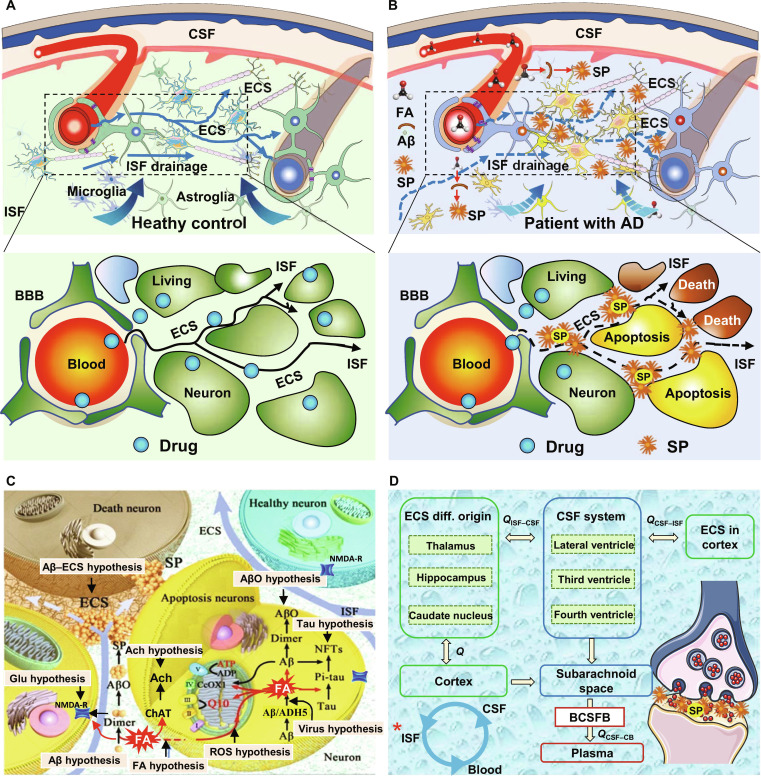
Aβ deposition and SP formation in patients with AD elicit ECS obstruction, compromise ISF clearance efficiency, and preclude targeted drug delivery to vulnerable neurons. (A and B) Comparative schematic of drug transport dynamics within the healthy versus pathologically remodeled cerebral ECS microenvironment in AD. (C) Pathological occlusion of the cerebral ECS culminates in the failure of therapeutic agents to reach their intended cellular targets. NMDA-R, *N*-methyl-d-aspartate receptor; Ach, acetylcholine; ChAT, choline acetyltransferase; ADH5, alcohol dehydrogenase 5; ATP, adenosine triphosphate; ADP, adenosine diphosphate. (D) Schematic illustration of substance trafficking routes from the cerebral ECS compartment to the systemic circulation.

Because of the deposition of Aβ in the brain ECS, the drainage of ISF slows or ceases, impairing the diffusion and clearance of substances within the ECS [82]. Concurrently, this pathological alteration in ECS not only slows the clearance of neurotransmitters from the synaptic cleft, leading to the accumulation of neurotransmitters such as acetylcholine and dopamine, but also exacerbates clearance impairments of toxic factors such as endogenous fractional anisotropy (FA) [102]. Endogenous FA is a key factor inducing Aβ aggregation to form SP and the formation of NFTs with tau protein [103]. The aforementioned pathological processes, including alterations in the brain ECS structure, neurotransmitter dysregulation, and accumulation of endogenous toxins, lead to impaired local synaptic transmission and neuronal cell death, ultimately causing cognitive and memory impairment in patients with AD. Incorporating the ECS theoretical framework into AD research provides a compelling explanation for the current therapeutic bottlenecks encountered in AD treatment. In the early stages of AD, when the ECS has not yet been completely blocked by Aβ, 5 clinical drugs for mild to moderate AD may still partially exert therapeutic effects, including 4 acetylcholinesterase inhibitors (donepezil, galantamine, rivastigmine, and tacrine) and one *N*-methyl-d-aspartate receptor antagonist [11,32,70,104,105]. However, in the late stages of AD, the blockage of the ECS by SP prevents these small-molecule polar chemotherapeutic agents from reaching the critical target areas of neuronal damage, thereby failing to exert neuroprotective effects or improve cognitive function. Therefore, for patients with AD at different stages of dementia, clinical treatment must consider the regional heterogeneity of ECS blockade/structural alterations and the impact of impaired ISF drainage on drug delivery. This represents a primary concern for novel drug development and clinical drug utilization. Therefore, future development of novel nanomedicines for AD should consider the entire process of drug entry through the BBB into ECS and delivery to therapeutic target sites, enabling targeted and specific drug design. For instance, ultrasound technology can be used to open the BBB and enhance drug penetration. Utilizing liposomal carriers to encapsulate and improve the BBB permeability of polar small-molecule drugs [106], along with further optimizing carrier surface charge, polyethylene glycol coating, and particle size, will facilitate drug delivery to the target site. In addition, as shown in Fig. 8 [12,107], the combined application of photobiomodulation and specific nanophototherapy combined with nanomedicines effectively disrupts Aβ plaques obstructing ECS, restores substance transport within the ECS and ISF flow, reduces endogenous FA accumulation, and restores cognitive function in AD rats. Of course, because of the discovery of the new ECS parcellation system [33,57], drug design strategies for AD and other common brain disorders must be recalculated on the basis of the translocation kinetics of drug molecules within the target ECS region to optimize the traditional PK [12].

### Incorporating substance transport and PKs of the ECS into the research for brain disease therapy

On the basis of the guidelines issued by the National Medical Products Administration and the International Council for Harmonization of Technical Requirements for Pharmaceuticals for Human Use, the administration frequency and dosage of systemic medications are determined according to the monitoring results of plasma drug concentrations [108,109]. However, this strategy cannot ensure that drugs reach the target neurons to exert their therapeutic effects. A primary reason for this historical theoretical gap is the lack of clinically applicable imaging analysis technologies for the human brain ECS, coupled with insufficient fundamental knowledge regarding the ECS. In the “The discovery of the brain ECS parcellation” section, we introduced the ECS parcellation system and clarified that small-molecule, water-soluble polar drugs present distinct PK profiles across different ECS compartments. Here, we further explore the importance and necessity of integrating ECS-related substance transport and PK into the research for brain disease therapy and analyze the technical support required for such systematic reform.

To illustrate this point, we take neuroprotective drugs for ischemic stroke as an example. In 2011, according to the Stroke Therapy Academic Industry Roundtable recommendations, a systematic analysis was conducted on the clinical development failures of neuroprotective drugs, identifying 7 critical design flaws responsible for these failures. Among the agents under clinical investigation at that time, citicoline was recognized as one of the most promising drug candidates [6]. Regrettably, citicoline was officially declared clinically ineffective in its phase III trial in 2012 [110]. Notably, neither the global roundtable summary nor subsequent analyses incorporated the critical role of the ECS into their assessments. A modest advancement has since been made: Prior to the clinical translation of brain-targeting therapeutics, regulatory authorities now recommend that researchers adopt drug concentrations in CSF, local ISF, or whole-brain homogenates as surrogate indicators to reflect the efficiency of brain drug delivery [111]. In reality, drug distribution within the brain ECS exhibits marked heterogeneity. On the basis of the arterial territory of the middle cerebral artery, infarct lesions in the middle cerebral artery occlusion (MCAO) model are predominantly localized in the Cn and its adjacent cortical regions. When citicoline is administered into the Tha region adjacent to the Cn, the drug fails to cross the ECS barrier to reach the ischemia-affected neurons and exert neuroprotective effects, even when the dosage and administration frequency are escalated to levels that induce neuroexcitotoxicity. In contrast, targeted delivery of neuroprotective agents to the upstream regions of ECS substance transport within ischemic areas, combined with dosing regimen optimization based on PK parameters (e.g., half-life and maximum distribution volume), yields a highly potent neuroprotective effect (Fig. 7). Specifically, in a rat model of prestroke, drug administration via ECS at a selected upstream site of Cn along the middle cerebral artery drainage territory achieved a 6-fold enhancement in neuroprotective coverage, using only 1/800 of the dosage required for intraperitoneal administration [11].

Technically, integrating the ECS into existing regulatory frameworks for evaluating the clinical efficacy of encephalopathy drugs demands technologies that enable accurate quantification of intracerebral drug distribution and provide PK/pharmacodynamic (PK/PD) data in target regions. Furthermore, imaging techniques that allow synchronous acquisition of structural and functional information on neurons, glial cells, microvessels, and the ECS in both animal and human brains will become indispensable. Currently, TB-MRI can provide PK parameters of small-molecule polar drugs in distinct ECS compartments [26]. Combined with AI technology, TB-MRI can also provide both the calculated results of diffusion rate and bulk flow velocity [35]. Moreover, by integrating the whole-body imaging capabilities of conventional MRI and radionuclide imaging, TB-MRI is technically well suited for conducting integrated PK/PD studies. Meanwhile, MDI offers an approach for synchronous visualization of neuroprotective drug effects on cells, blood vessels, and the ECS during clinical evaluations [50]. This synchronous acquisition technology also yields noninvasive biomarkers for assessing drug effects on different compartmental structures and intercompartmental interactions, serving as a practical tool for exploring disease mechanisms and the pathways underlying drug action.

### ECS-targeted therapeutic strategies for brain diseases

On the basis of the aforementioned research findings, future drug design and treatment strategies should consider brain ECS as a critical factor in neurological diseases. We have summarized the pathological changes related to ECS in various brain disorders. A challenge in understanding ECS pathophysiology lies in distinguishing whether changes in ECS structure and function are primary drivers of brain disease. This distinction holds critical guidance for formulating treatment strategies. Increasing evidence suggests a dynamic bidirectional regulatory relationship: In the early stages of disease, mild ECS dysfunction may initiate pathological cascades (for instance, impaired toxic metabolite clearance precedes protein aggregation); whereas, in later stages, disease-specific pathological mechanisms actively remodel ECS structures, establishing self-perpetuating pathological cycles. This conceptual framework provides essential theoretical background for interpreting the specific ECS changes associated with different diseases, as well as direction for developing stage-specific therapeutic interventions. So far, no preclinical or clinical study has systematically integrated the ECS into its research framework. Given that the ECS undergoes pathological changes during disease progression and serves as a core pathway for drug delivery and neuromodulation, this research field is particularly demanded. Various ECS-targeted intervention strategies have demonstrated remarkable efficacy in preclinical models, offering broad prospects for future treatments of CNS diseases.

#### Alzheimer’s disease

The typical pathological changes and neural damage mechanisms of AD include protein homeostasis imbalance, dysregulated immune responses, and abnormal neural circuitry, among which the abnormal metabolism of Aβ represents a key initiating event. Soluble Aβ oligomers within the ECS trigger early neurotoxicity and cognitive deficits by disrupting the transport of synaptic receptors (*N*-methyl-d-aspartate and AMPA receptors) and impairing long-term potentiation [112,113]. Dysregulation of the biophysical microenvironment of the ECS constitutes a key factor driving AD progression, characterized by alterations in ECS spatial conformation and fluid dynamics related to ISF drainage, as elaborated in the “Incorporating pathological ECS changes into research framework of brain diseases” section [28,114]. These pathological changes compromise the substance transport function of the ECS, reduce the brain’s network integration capacity, and disrupt memory formation. Given the aforementioned ECS-related pathological alterations, the research advances in ECS-targeted therapeutic strategies for AD are elaborated in the subsequent section.

PBMT uses visible/NIR nonionizing radiation to induce photochemical reactions and physiological changes in target tissues. RL (630, 632.8, and 635 nm) and NIR (808, 1,070 nm, 4 J/cm^2^, 10 Hz) reduce Aβ deposition [81,115,116], up-regulate brain-derived neurotrophic factor (BDNF) [117], and improve cognitive function in dementia mice [81,115]. In vitro, 810 nm NIR alters microtubulin secondary structure (decreased α helix and increased β sheet) [118]. Notably, 630-nm RL penetrates the skull, activates formaldehyde-degrading enzymes to enhance FA clearance, thereby reducing Aβ deposition, promoting ISF drainage, remodeling ECS structure, and improving cognition in AD models [116,119].

FUS combined with microbubbles regulates ECS via sonoporation. At low sound pressure, microbubbles induce steady cavitation, reversibly opening the BBB through vascular wall shear forces to facilitate antibody drug delivery [91,120]. FUS increases ECS volume fraction, reduces tortuosity, and decreases solute diffusion resistance [121]. It also reduces amyloid positron emission tomography signals, indicating Aβ clearance potential [122].

TMS modulates neural networks via electromagnetic induction. High-frequency stimulation (>5 Hz) up-regulates the BDNF–Tropomyosin-related kinase B (BDNF-TrkB) pathway to promote synaptic regeneration [123]. By regulating default mode network functional connectivity, it reverses Aβ-induced network collapse [124–126]. In addition, 40-Hz gamma transcranial ac stimulation activates microglia, induces vasodilation and slow-wave oscillations, and enhances Aβ clearance [127].

Mesenchymal stem cells, neural stem cells, and induced pluripotent stem cells remodel the ECS microenvironment via paracrine effects and immune regulation. Transplanted stem cells secrete anti-inflammatory factors (transforming growth factor-β and interleukin-10 [IL-10]) and neurotrophic factors (BDNF and vascular endothelial growth factor [VEGF]) to promote neurogenesis and synaptic remodeling. Stem-cell-delivered microRNAs (e.g., miR-146a) reduce Aβ production/aggregation by modulating inflammation [128–130]. Notably, dense ECM in the pathological microenvironment increases ECS tortuosity, forming a physical barrier that restricts transplanted cell migration, homing, distribution, and survival [131,132].

Deep cervical lymphovenous anastomosis (LVA) intervenes in the glymphatic system via microsurgical anastomosis of high-resistance deep cervical lymphatics to the low-pressure cervical venous system. This enhances CSF–ISF exchange and drainage, accelerating clearance of neurotoxic metabolites (Aβ and phosphorylated tau) [133–135]. Clinical studies confirm its safety and feasibility, with short-term improvements in mini-mental state examination scores and reduced CSF AD biomarkers; however, its effect on complex cognition (e.g., Montreal cognitive assessment scores) remains unclear [136,137]. High-quality randomized controlled trial evidence supporting LVA for AD is currently lacking [138,139]. Exogenous VEGF-C improves meningeal lymphatic function, enhances Aβ clearance, and ameliorates cognition in AD models [140,141].

Collectively, physical, surgical, and cell-based strategies targeting the ECS hold substantial potential for AD therapy by improving substance transport and remodeling the brain microenvironment.

#### Ischemic stroke

Neuronal damage following ischemic attack progresses cascadingly because of reduced or obstructed cerebral perfusion, triggering energy failure, ionic imbalance in the ischemic core, glutamate-mediated excitotoxicity in the penumbra, and reactive oxygen species (ROS)-induced oxidative stress, inflammation, and BBB disruption during reperfusion, ultimately leading to neuronal apoptosis or pyroptosis [142,143]. Current therapies (intravenous thrombolysis, endovascular thrombectomy, neuroprotective agents, and stem cell therapy) primarily focus on damaged neurons or neurovascular units but overlook ECS pathology [144,145]. Consequently, traditional therapeutic strategies not only need to overcome the challenge of drug entry into the brain through the BBB via systemic administration but also address the difficulty of drugs reaching damaged neurons within the ECS after crossing the BBB. The ECS provides a novel perspective and potential solutions to these challenges.

To enhance the drug delivery to the brain to treat ischemic stroke, Bobo et al. [146] proposed the convection-enhanced delivery (CED) technique for direct intraparenchymal administration in the 1990s. This technique assumes that the brain is a solid tissue and drives substance transport in the brain parenchyma through pressure, thereby bypassing the hindrance of the BBB [146]. Pressure-induced backflow is the major technical obstacle of CED [147]. To address this, Han’s research team applied machine-learning-aided TB-MRI and confirmed that ECS drug molecule transport is primarily diffusion-dominated [35]. They also proposed a concentration-driven drug delivery method of simple-diffusion delivery (SDD), which bypasses the BBB to achieve efficient, low-toxicity neuroprotection, providing a novel therapeutic paradigm via ECS for ischemic stroke, brain tumors, and other brain diseases [11].

Before the ECS was incorporated into the mechanistic research of encephalopathy treatment, the therapeutic mechanism of encephaloduroarteriosynangiosis (EDAS) for moyamoya ischemic disease remained unclear, which seriously hindered the promotion and application of this surgery in the treatment of ischemic stroke. Previous mechanistic studies on EDAS have primarily focused on blood vessels, failing to adequately explain the early clinical improvement of stroke symptoms. Recently, Lian et al. [43] applied a modified EAI (m-EAI) and confirmed in a rat MCAO reperfusion model that the mechanism of this surgical treatment for cerebral ischemic injury lies in accelerating substance transport within the brain tissue ECS. By promoting the clearance of neurotoxic factors such as inflammatory factors in the penumbra, favorable neuroprotective effects can be achieved: Arterial pulsation synchronously enhances ECS transport and facilitates clearance of toxic factors from the penumbra, significantly reducing infarct volume (from 23.71% to 7.69%) and alleviating cerebral edema (brain water content: 79.68% in the m-EAI group versus 83.52% in the transient MCAO group) [43,148].

Similarly, application of 755-nm NIR light to the MCAO drainage region achieves a more pronounced acceleration of ECS molecular diffusion and ISF drainage compared with m-EAI. This intervention promotes the clearance of inflammatory factors and pathological proteins, mitigates cerebral ischemia–reperfusion injury, improves poststroke cognitive impairment, and reduces infarct volume by 45.3% on day 28 posttreatment [44].

#### Parkinson’s disease

PD is characterized by progressive degeneration of dopaminergic neurons in the nigrostriatal pathway, driven by mitochondrial dysfunction, α-synuclein (α-syn) aberrant aggregation, and amplified oxidative stress [149,150]. These pathological changes disrupt the distribution of dopamine, glutamate, and other neuroactive substances in the striato–cortical–thalamic circuit, leading to cardinal motor symptoms such as bradykinesia and tremor [151]. Current therapies include dopaminergic pharmacotherapy [152], neuroprotective agents [150], gene therapy [153], cell transplantation [154], noninvasive neuromodulation [155,156], and surgical interventions (lesioning and DBS) [157] but with obvious limitations: Dopaminergic medications lose efficacy and induce motor complications; noninvasive approaches lack spatial resolution and penetration depth; DBS has variable responses with unclear mechanisms; crucially, existing treatments fail to coordinate ECS homeostasis, neurochemical balance, and neuronal protection. Accumulating evidence identifies ECS dysfunction as a critical contributor to PD pathogenesis: Structurally, PD rat models show significant changes in ECS *α* and *λ*, with increased tortuosity following α-syn oligomer-induced neurodegeneration [158,159]; functionally, ISF drainage is impaired in PD rats (especially in the substantia nigra and Cn), which can be partially reversed by levodopa treatment [160–163].

ECS modulation has been validated as an effective therapeutic strategy for PD. Mechanical vibration, photostimulation, and electromagnetic approaches can alter ECS architecture and molecular transport to improve brain function: Low-intensity FUS (LIFUS) expands ECS and PVS to facilitate small-molecule diffusion [94], and combining LIFUS with intravenous microbubbles enhances deep brain molecular transport without damaging the BBB [91]. Our previous work showed that high-frequency (130-Hz) subthalamic nucleus (STN)-DBS remotely modulates ECS structure and ISF drainage in the substantia nigra of PD rats (reducing effective diffusion coefficient D* by 11.2%, shortening half-life *T*_1/2_ by 15.7%, and increasing clearance coefficient *k′* by 23.7%). Mechanistically, STN-DBS up-regulates HA content and AQP4 expression, restores excitatory amino acid transporter 2-mediated glutamate transport, accelerates ISF drainage and α-syn clearance, ultimately protects dopaminergic neurons [164]. These studies highlight the pivotal role of ECS dysfunction in PD and validate a therapeutic framework targeting ECS-mediated α-syn clearance, laying theoretical and technical foundations for ECS as a novel PD treatment target.

#### Glioma

Gliomas are the most common primary malignant tumors of the CNS [165,166]. Gliomas typically exhibit expanded ECS volume and increased tortuosity, which are closely correlated with pathological grades: Low-grade gliomas have an *α* value of 0.29, whereas high-grade gliomas show an *α* value as high as 0.44 and a *λ* value increased to 1.78 (compared with normal brain tissue) [167]. The overexpressed tenascin in the ECM, combined with interstitial fibrosis and vascular dysfunction, severely restricts the transportation of the neuroactive substances and therapeutic drug agents [168]. Current clinical treatments mainly include surgery, radiotherapy, chemotherapy, and biotherapy. CED was approved by FDA for malignant glioma treatment in 2009 [146,169,170]. However, insufficient understanding of ECS substance transport anisotropy and hydrodynamic laws in clinical practice leads to a series of critical technical bottlenecks, including drug backflow, uncontrollable distribution range, and inability to perform precise quantitative therapy on tumor cells.

The application of TB-MRI enables real-time monitoring of ECS substance transport, which is crucial for addressing technical limitations of CED. Notably, tumor growth significantly alters the pathways and velocities of ECS substance transport [168]. More importantly, machine-learning-based analysis of TB-MRI results has revealed that diffusion is the primary form of molecular transport in deep brain regions. Therefore, the concentration-driven SDD method is expected to be more efficient than CED for chemotherapeutic drug delivery via the ECS pathway, providing a novel direction for precise glioma therapy.

#### Multiple sclerosis

Multiple sclerosis (MS) is a chronic immune-mediated CNS demyelinating disorder characterized by multifocal demyelination, axonal injury, neurodegeneration, and heterogeneous progressive neurological deficits from autoreactive immune cell infiltration, myelin destruction, and impaired signal transmission [171]. Recent studies confirm brain ECS functional abnormalities as key neural injury mechanisms: Demyelination disrupts the ECS parcellation barrier, triggering abnormal striatal ISF drainage into lesioned regions (striatal ECS structure unchanged but drainage half-life shortened by ~8%, reversible upon remyelination), and causing aberrant neuroactive substance distribution (especially in purine, arginine, and proline metabolism pathways), forming a “demyelination–ECS dysregulation–pathological accumulation–neural injury” vicious cycle [33,58]. Current MS therapies (immunomodulatory and remyelination-promoting, neuroprotective, and symptomatic care) have substantial limitations despite indirect ECS associations: lack of ECS as a core target (failing to block the pathological cascade), confined indirect effects (direct roles in ECS barrier repair/ISF drainage unclarified), imbalanced synergistic regulation (no targeted intervention for ECS-induced metabolic abnormalities), and absence of precise ECS function assessment systems [58].

Han et al. first confirmed the efficacy of ECS pathway-based treatment for MS, providing novel precise targeting strategies. Using a unilateral demyelination rat model and TB-MRI, they verified the critical pathological role of ECS parcellation barrier disruption and abnormal ISF drainage and found that remyelination can reverse ECS functional abnormalities [58]. Further research revealed that metabolic pathway abnormalities induced by ECS barrier impairment are directly correlated with inflammation and neural injury, laying a theoretical foundation for targeted modulation of the ECS metabolic microenvironment to block pathological progression. Future research should focus on strengthening the exploration of ECS targeting mechanisms, developing ECS-targeted intervention approaches, and establishing multitarget synergistic regimens combining ECS regulation with existing immunomodulation and remyelination-promoting and neuroprotective therapies to improve the MS treatment system.

#### Schizophrenia

The pathophysiological mechanisms of schizophrenia (SCZ) remain incompletely elucidated, with mainstream hypotheses focusing on neural circuit dysfunction (dopaminergic hyperactivation and glutamatergic hypoactivity), synaptic dysfunction, and chronic neuroinflammation [172–176]. Recently, ECS integrity has been recognized as a critical regulatory factor: AQP4 gene polymorphisms linked to SCZ risk disrupt CSF–ISF exchange and prolong inflammation [177,178]; enlarged choroid plexus in patients with somatic symptom disorder impairs glymphatic clearance and exacerbates symptoms [179–181]; neuroimaging shows reduced perivascular ECS diffusion and imbalanced vasculature–CSF coupling in patients with SCZ [182–186]. Current treatments (antipsychotics, electroconvulsive therapy, and rTMS) target cellular/neural circuit levels, with limitations including neglect of extracellular microenvironment pathology and low drug delivery efficiency due to BBB/ECS obstructions, leading to 30% treatment-resistant cases with persistent cognitive impairments [187].

ECS-based targeted interventions offer a promising direction for SCZ therapy. ECS-mediated precise diffusion drug delivery can bypass BBB constraints; under tracing technique guidance, lower doses can achieve better drug enrichment in target brain regions, potentially enhancing efficacy and reducing adverse effects. AQP4 modulators and anti-inflammatory drugs are expected to restore ECS fluid transport function and alleviate inflammation-related obstructions, providing new ideas for improving negative symptoms and cognitive dysfunction. To advance relevant research systematically, the following optimizations are needed: (a) focus on core SCZ populations to explore the potential applications and mechanisms of ECS regulatory technologies; (b) investigate the combination of ECS-targeted strategies with existing treatments; (c) strengthen basic mechanistic research to clarify the interactions between ECS pathological changes, neural circuits, and the BBB.

#### Epilepsy

Epilepsy is a chronic neurological disorder characterized by abnormal synchronous neuronal discharges and recurrent seizures, with a pathophysiological core of excitation–inhibition imbalance that drives global network oscillations [188,189]. Postepisode plasticity changes and ion homeostasis disruption form a disease progression vicious cycle [190–192]. Currently, antiepileptic drugs are first-line therapy (controlling seizures in ~70% patients), with surgical resection and neuromodulation (e.g., vagus nerve stimulation and DBS) as complements for drug-resistant cases [193]. However, therapeutic bottlenecks are increasingly linked to the ECS “physical barrier” hypothesis and diffusion parameter alterations [28]. Under chronic conditions (e.g., temporal lobe epilepsy and status epilepticus), reactive astrocyte proliferation and pathological ECM deposition drastically reduce epileptogenic cortex volume fraction (~30% reduction) with increased tortuosity and structural collapse [194]. These changes impede drug diffusion, delay excitatory substance (glutamate and K^+^) clearance (local K^+^ >10 mM) to intensify synchronous discharges via electrical field coupling, reduce ECS efflux efficiency (e.g., abnormal AQP4) causing neurotoxic metabolite accumulation, and interact with seizure-generated ROS/IL-1β to disrupt ECS integrity, forming a hyperexcitable microenvironment that sustains disease progression [195–199].

Novel ECS-targeted therapeutic strategies emphasize “environmental remodeling”, aiming to bypass synaptic drug resistance by restoring ECS volume and ion homeostasis to block cascade reactions. Emerging technologies offer new approaches to overcoming ECS physical barriers: (a) Pulsed ultrasound modulates neural excitability and temporarily mechanically dilates ECS and PVS to enhance drug penetration and metabolite clearance [200]; (b) CED uses pressure gradients to deliver drugs directly into interstitial space, bypassing BBB and convoluted ECS [201]; and (c) nanocarriers traverse convoluted ECS to precisely deliver drugs to epileptogenic zones while evading efflux transporters [202]. In addition, Na–K–2Cl cotransporter isoform 1/K–Cl cotransporter isoform 2-targeted interventions (e.g., furosemide and bumetanide) suppress glial swelling and reverse ECS contraction, blocking induced discharges in animal models and suppressing interictal spikes clinically with reported 50% seizure frequency reduction [203,204]; bumetanide phase II studies showed 70% seizure reduction, with ongoing BBB penetration optimization [205]. Restoring ECS volume via high-molecular-weight HA supplementation or osmotic adjustment (e.g., mannitol) is recommended as intraoperative/emergency strategy in international guidelines, with observational inhibition rate up to 80%.

## Conclusion and Future Directions

With advances in imaging detection technologies, the integration of ECS into the formal brain science framework has progressively matured. However, the incorporation of ECS into the research system of neuroscience and brain disease treatment requires efforts across multiple dimensions. Precise and effective treatment of brain diseases necessitates comprehensive elucidation of their pathogenic and progressive processes and mechanisms, which, in turn, requires the enhancement and development of cross-scale structural information processing and analytical capabilities. As a key component of the brain ISS, the processes of substance transport within the ECS and its drainage back to the systemic circulation are intricate, requiring multimodal imaging technologies to cover all drainage pathways and their regulatory mechanisms.

To establish a research system compatible with nanoscale ECS and microscale microvessels and neural cells, it is essential to develop synchronous information acquisition technologies and explore the application of simulation modeling to clarify the complex interactions among neurons, glial cells, ECS, and blood vessels. Advancement of cross-scale, multimodal brain detection technologies is imperative to fully reveal the mechanisms of brain diseases and more rationally facilitate the preclinical research and development design of novel drugs and medical devices.

A pivotal step in translating ECS research into clinical practice is establishing human brain imaging and measurement systems for precise ECS quantification [8,16,18,23,50]**.** Both mechanistic studies of brain disorders and therapeutic efficacy assessments require technologies that can simultaneously measure cellular, extracellular, and vascular compartments. Adopting a “cell–ECS–vasculature” framework holds promise for comprehensively understanding the onset, progression, and outcome of neurological diseases [8,11,101,206–208]. In multicenter clinical trials, relying on multicompartment imaging technology for human brain and embedding ECS parameters into regulatory assessments will enable the optimization of drug design and delivery strategies and establish a new framework for early-stage screening of compounds with superior spatial efficacy and safety.

The historical neglect of the brain ECS stems fundamentally from limitations in early scientific understanding and investigative methodologies, which precluded the full recognition of its structural independence and functional significance. Consequently, the ECS was excluded from formal medical education curricula, with conventional courses in neuroanatomy, neurophysiology, neuropathology, and neuropharmacology rarely acknowledging it as a distinct structural entity. Now that modern research has firmly established the ECS as a critical component of brain architecture with essential physiological roles, integrating ECS-related knowledge into existing textbooks and embedding the “cell–ECS–vasculature” model into both research paradigms and instructional frameworks will fundamentally reshape our understanding of the brain and neurological disorders [16–18,35,50]. Furthermore, the advancement of cross-scale, multimodal brain imaging and measurement technologies is indispensable for fully elucidating the complex interactive dynamics among neurons, glial cells, the ECS, and blood vessels. Against this backdrop, the establishment of a structurally complete, ECS-integrated neuroscience paradigm has become a highly anticipated goal for the entire field.

## Data Availability

The data supporting the findings of this review are available from public domains and clinical trial databases. Specifically, the clinical trial data for AD and PD presented in Fig. 1 were retrieved from ClinicalTrials.gov (https://clinicaltrials.gov/). The bibliometric analysis data presented in Fig. S1 were derived from the Elsevier Scopus database.
